# Nerve growth factor has a modulatory role on human primary fibroblast cultures derived from vernal keratoconjunctivitis-affected conjunctiva

**Published:** 2007-06-21

**Authors:** Alessandra Micera, Alessandro Lambiase, Barbara Stampachiacchiere, Roberto Sgrulletta, Eduardo Maria Normando, Sergio Bonini, Stefano Bonini

**Affiliations:** 1 CIR Laboratory of Ophthalmology, University Campus Bio-medico, Rome, Italy; 2 IRCCS-G.B. Bietti Eye Foundation, Rome, Italy; 3 Second University of Naples & Institute of Neurobiology and Molecular Medicine, National Research Council (INMM-CNR), Rome, Italy

## Abstract

**Purpose:**

To evaluate the role of nerve growth factor (NGF) in remodeling processes of vernal keratoconjunctivitis (VKC). VKC is a chronic inflammatory disorder of the conjunctiva and is characterized by marked tissue remodeling. NGF, a pleiotrophic factor with documented profibrogenic activities, is produced by inflammatory and structural cells populating the VKC conjunctiva and is increased in the serum and tears of VKC patients.

**Methods:**

Primary cultures of VKC-derived fibroblasts (VKC-FBs) were exposed to increasing NGF concentrations (1-500 ng/ml) to evaluate and compare the expression of α-smooth muscle actin (αSMA, a defining myofibroblast marker), collagens (types I and IV), and metalloproteinases and tissue inhibitors (MMP9/TIMP1, MMP2/TIMP2) at the biochemical as well as molecular levels.

**Results:**

Endogenous NGF was increased in the VKC-FB supernatant, as compared to healthy-FB supernatant. VKC-FBs expressed αSMA and increased types I and IV collagens. VKC-FBs, and in particular all αSMA positive cells, expressed both trkA^NGFR^ and p75^NTR^, while healthy-FBs only expressed trkA^NGFR^. Exogenous NGF did not change αSMA expression, while αSMA expression was enhanced by specific neutralization of p75^NTR^. NGF (10 ng/ml) exposure significantly decreased type I collagen expression, without affecting type IV collagen, and increased MMP9mRNA and protein.

**Conclusions:**

The autocrine modulation of differentiation and response of VKC-FBs to NGF exposure with downregulation of type I collagen and upregulation of MMP9 expression supports a relevant role for NGF in tissue remodeling of VKC.

## Introduction

Vernal keratoconjunctivitis (VKC) is a severe chronic inflammatory disease of the conjunctiva. The disease is characterized by an allergic phenotype with infiltration of lymphocytes (mostly Th2), eosinophils, and mast cells [1]. In VKC, Th2 inflammation is associated with marked tissue remodeling as shown by fibroblast (FBs) activation, epithelial growth, subepithelial fibrosis, and extracellular matrix (ECM) deposition, resulting in giant papillae formation [2]. ECM analysis has shown a substantial increase in total collagen (procollagen and types I, III, and IV collagen), with an altered collagen ratio, both in tears and conjunctiva [3].Increased ECM is referred to increased expression of cytokines and growth factors, from either Th2-type and other inflammatory cells, which are known to stimulate resident/activated FBs to overproduce ECM [4]. Overt production and deposition of ECM eventually results from an unbalance between collagen production and Matrix Metalloproteinases (MMP1, MMP2 and MMP9) activities [1,3].

A variety of inflammatory- and stromal-derived cytokines, such as TNF-α, IL-1, IL-4, IL-13, and IL-6, and growth factors, such as transforming growth factor β1 (TGFβ1) have been reported to cause chronic inflammation, structural changes, tissue remodeling, as well as fibrosis [5,6]. All these factors are increased in the peripheral blood, tears, and conjunctiva of patients with VKC [2].

To date, TGFβ1 represents the main profibrogenic factor responsible for the imbalance in ECM metabolism as well as for the chemoattraction and survival of FBs and myoFBs. Interestingly, nerve growth factor (NGF) has been recently prospected as another factor playing a role in stromal-epithelial interaction during wound-healing and tissue repair processes [7,8]. Certainly, NGF is significantly increased in both serum and conjunctiva of patients with VKC [9].

We previously reported a profibrogenic NGF effect on in vitro primary cultures of healthy conjunctival FBs (healthy-FBs) [8]. Since nothing is known about the possible role of NGF (increased in VKC-affected conjunctiva) on primary cultures of VKC conjunctival FBs (VKC-FBs), we exposed these cells to increasing NGF concentrations. In addition, some in vitro parameters of tissue remodeling were evaluated.

## Methods

### Reagents

Unless otherwise noted, sterile tissue culture plastic-ware and analytical grade reagents were from NUNC (Roskilde, Denmark), SERVA (Weidelberg, Germany), ICN (Costa Mesa, CA), Euroclone (Milan, Italy), and Invitrogen-Gibco (Paisley, UK).

### Cell culture and experimental procedure

FBs were isolated from the upper tarsal VKC (n=3; 3M, range 11-15, mean age 12.67±2.08) and sex/age matched healthy (n=3) conjunctiva. Informed consent was given by the parents of each patient and the approval of the Intramural Ethic Committee was granted, in conformity to the Declaration of Helsinki. The diagnosis of active VKC was based on history, clinical examination and the presence of eosinophils in conjunctival biopsy. Clinical scores (0-3: 0, absent; 1, weak; 2, mild; 3, severe) for each ocular symptom (itching, tearing, photophobia and foreign body sensation) and each sign (conjunctival hyperemia, mucous discharge, papillae and corneal epithelial erosion) were assigned at the time of examination. Total symptom (range 0-3) and sign scores (0-12) were calculated showing: total symptom score of 7.33±4.51 and total sign score of 8.67±3.05. The three patients showing lid-ptosis were submitted to surgical removal of the papillae that were processed in this study.

Biopsies were put as explants in 24-well plates and left to attach for 10 min, before adding medium (DMEM) supplemented with 10% heat-inactivated Fetal Bovine Serum (FBS), 2 mM glutamine, 100 U/ml penicillin and 100 μg/ml streptomycin. Cells outgrowing from biopsies (37 °C, 5% CO_2_ in air, by 1 week), were quickly trypsinized (0.2% trypsin/0.025% EDTA), assessed for the absence of epithelial cell contaminants (cytokeratin 19, 1:100; Dako Corp., Carpinteria, CA) and screened as mycoplasm free cultures (otherwise treated for 1 generation with 5 μg/ml Mycoplasm Removal Agent; ICN). FBs were sub-cultured (T-21/T-75 cm^2^ flasks) and used for the experiments (3rd to 4th passage) after 3 h of serum starvation (synchronization and reduction of autophosphorylation) [10].

For the specific experiments, confluent FBs were treated with increasing NGF concentrations (murine βNGF; 1-500 ng/ml; produced according to a standardized protocol [11]) in minimal DMEM (0.5% FBS) [8]. Biochemical and molecular evaluations were performed 2 days after stimulation. For blocking experiments, serum-starved FBs were preincubated for 1 h with 500 ng/ml αNGF (R&D Systems, Minneapolis, MN) or with 100 ng/ml αtrkA^NGFR^ (Calbiochem, La Jolla, CA) or 100 ng/ml αp75^NTR^ (Calbiochem) specific neutralizing antibodies, before the addition of NGF alone (untreated FBs) or in the same blocking reagents (pretreated FBs), for two days. Whenever required and according to literature [6,12], human TGFβ1 (1-10 ng/ml, R&D) was used as positive control in some experiments.

### Confocal microscopy

Confluent monolayers (on sterilized round glasses, EMS, Hatfield, PA) were washed in Hank's Balanced Sodium Salt (HBSS), fixed in 2% ρ-formaldehyde (PFA) - 0.1 M Phosphate Buffer (PB), rinsed in 10 mM PB - 137 mM NaCl (phosphate buffered saline, PBS), quenched in 50 mM NH_4_Cl-PBS, permeabilized in 0.5% Triton X-100 PBS (TX-PBS) and finally blocked in 0.8% bovine serum albumin (BSA)-PBS. Monolayers were then probed with the following antibodies in 0.05% Tween20-PBS (TW-PBS): rabbit anti-human trkA^NGFR^ antibody (2 μg/ml; Santa Cruz Biotech, Santa Cruz, CA); goat anti-human p75^NTR^ antibody (2 μg/ml; Santa Cruz) and mouse anti-human αSMA antibodies (1/50; Novocastra, Milan, Italy). Specific binding of the primary antibody was detected using Cy2 or Cy3 conjugated F(ab)_2_ antibodies in TW-PBS (Jackson Laboratories, West Grove, PA). Control immune-staining was performed by substituting primary antibodies with control irrelevant IgG (Vector Laboratories, Burlingame, CA). Glasses were mounted on slides using an anti-fade medium (Vectashield, Vector) and viewed with a confocal inverted microscope (E2000U; Nikon, Tokyo, Japan). Images and brightness/contrast levels were captured/evaluated using the C1 software (Nikon) and the Adobe Photoshop 7.0 program (Adobe Systems Inc., San Jose, CA), respectively.

### Relative real-time RT-PCR

Total RNA (1x10^6^ cells) was extracted in OMNIzol (Euroclone), DNaseI treated (AB1709; Ambion Inc., Austin, TX), quantified (λ_260_/λ_280_ >1.8) and checked for RNA integrity. Three μg total RNA were reverse transcripted (Mu-MLV; Finnzyme, Milan, Italy) in a PTC-100 programmable thermocycler (MJ Research, Watertown, MA). cDNAs (3 μl for target gene and 1 μl for referring gene) were amplified using the Opticon2 real time termocycler (MJ Research) in a 20 μl final volume of SYBR Green PCR mixture (Applied Biosystems, Foster City, CA). The temperature profile included initial 95 °C for 15 min incubation, followed by 35-47 cycles of denaturation at 95 °C for 30 s, annealing at 55-60 °C for 25 s (see previous paragraph for specific Ta), elongation at 72 °C for 30 s, fluorescence monitoring at 60-90 °C, 0.01 °C for 0.3 s, and further incubation at 72 °C for 5 min. PCR amplification primer pairs were received from Genbank or Primer 3 (MWG Biotech, Ebersberg, Germany), were as follows: NGF (forward: CTG GCC ACA CTG AGG TGC AT; reverse: TCC TGC AGG GAC ATT GCT CTC; 120 bp; 53 °C Ta; Genbank BC011123); trkA^NGFR^ (forward: CAT CGT GAA GAG TGG TCT CCG; reverse: GAG AGA GAC TCC AGA GCG TTG AA; 102 bp; 58 °C Ta; Genbank M23102); p75^NTR^ (forward: CCT ACG GCT ACT ACC AGG ATG AG; reverse: TGG CCT CGT CGG AAT ACG; 147 bp; 57 °C Ta; Genbank AF187064); αSMA (forward: GAA GGA GAT CAC GGC CCT A; reverse: ACA TCT GCT GGA AGG TGG AC; 125 bp; 60 °C Ta; Genbank BC017554); MMP-9 (forward: CAG TCC ACC CTT GTG CTC TTC C; reverse; GCC ACC CGA GTG TAA CCA TAG C; 113 bp; 60 °C Ta; Genbank BC006093) and the referring GAPDH gene (forward: GAA GGG GTC ATT GAT GGC AAC; reverse: GGG AAG GTG AAG GTC GGA GTC; 100 bp; 53 °C Ta; Genbank BC013310). Product specificity was assessed by melting curve analysis of each sample carried out in duplicate as well as by gel size-fractioning. C_t_ values normalized samples showing good melting curves were used for statistical analysis. Differences in PCR product expression were evaluated by REST© software [13].

### Proliferation assays

The effect of increasing NGF concentration on VKC-FB proliferation was investigated by counting the cells, after brief enzymatic digestion, using the trypan blue exclusion test. The expression of the nuclear proliferating factor ki67, recognizing all the cell cycles except G0 [14], was investigated on previously fixed monolayers stained with rabbit antihuman ki67 antibody (1:1000, Santa Cruz) and developed by fluorescent ABC technique (Vector).

### Nerve growth factor ELISA

To evaluate NGF in the supernatants of VKC and healthy FBs, we carried out a a two-site NGF ELISA (sensitivity=0.5 pg/ml) [15]. In brief, 96-well Maxisorp ELISA plates were precoated with monoclonal antihuman βNGF antibodies (0.4 μg/ml; MAB256, RαD). Both standards (0.15 pg/ml to 1 ng/ml βNGF; Alomone, Jerusalem, Israel) and samples (1/4) were incubated at 4 °C for 18 h. ELISA was developed by using polyclonal biotinylated antihuman ηNGF antibodies (0.15 μg/ml, 500-P85Bt; Peprotech, Milan, Italy), HRP-streptavidin (1/300; DY998, RαD) and the ready-to-use TMB substrate (Zymed, San Francisco, CA). Optical density (OD) was measured at λ_450-550_ by a microplate ELISA reader (Sunrise, Tecan Systems, Inc., San Jose, CA). The biological activity of FB-derived NGF was tested separately by using a standardized PC12 bioassay [16]. Protein normalization was achieved by a Bio-Rad protein assay, using BSA equivalent as a standard (Bio-Rad Laboratories, Inc., Hercules, CA).

### Cell surface ELISA

To investigate the effect of NGF on αSMA expression by VKC-FBs, an ELISA was carried out on monolayers [17]. Briefly, FBs (5x10^5^/0.20 ml) were seeded on 96-well plates. Once the cells reached confluence, they were incubated with increasing NGF concentrations. After culturing, plates were washed in HBSS containing 0.1% CaCl_2_, PFA post-fixed, quenched for endogenous peroxidases (0.3% H_2_O_2_ in PBS for 15 min), permeabilized in TX-PBS, and dehydrated with 95% ethanol before the assay. The plates were subsequently processed for quantification of αSMA (1/500, Novocastra), types I and type IV collagen (1/1000; Santa Cruz) proteins, using monoclonal and polyclonal specific antibodies diluted in TW-PBS. Biotin-conjugated antimouse or antigoat antibodies and HRP-streptavidin (1/10000 and 1/7000, respectively; Zymed) specific binding were developed with a ready-to-use TMB and quantified using an ELISA reader.

### Western blot analysis

Total proteins were extracted by 30 min incubation with cold lysis-buffer (50 mM Tris-HCl pH 7.5, 150 mM NaCl, 1 mM EDTA, 1% Nonidet P-40, 0.1% SDS, 7 μg/ml aprotinin, and 1 mM PMSF). Equivalent protein amounts (40-80 μg; Bio-Rad protein assay) were boiled for 5 min under reducing conditions and fractionated on a 4% stacking/6-15% gradient resolving gel at 160 V/60 min (Miniprotean3 apparatus, Bio-Rad). Electrophoresed proteins were transferred to Hy-bond membranes using a semi-dry blotting apparatus (12 V/45 min, Bio-Rad). A molecular weight marker (6-210 kDa; SERVA) was run in parallel. Transferred proteins (Ponceau S staining) were probed at 4 °C for 18 h with primary antibodies diluted in TW-PBS (αSMA, types I and type IV collagen: 0.2 μg/ml; MMP9: 0.4 μg/ml), labeled with secondary POD-conjugated specific antibodies (1/7000, 90 min; Jackson), and developed by ECL technique (SuperSignal West Pico Trial; Pierce, Rockford, IL) in a high performance Kodak imager station (Kodak 550, Eastman Kodak Company, Scientific Imaging Systems, Rochester, NY). Bands were digitally captured using the 1D Kodak Image Analysis Software, subjected to densitometric analysis, and processed in Adobe Photoshop 7.0. Membranes were stripped at 56 °C for 45 min in 1 M Tris-HCl containing 2% SDS and 1.25 mM β-mercaptoethanol and reprobed with GAPDH antibodies (0.2 μg/ml; Abcam, Cambridge, UK), to verify equal protein loading.

### SDS-PAGE zymography

After culturing, the conditioned media were collected and clarified by centrifugation. MMP activity was analyzed by zymography, using a procedure described in reference [18]. Briefly, 50 μl conditioned media were mixed with SDS sample buffer, without β-ME, and heated for 37 °C/30 min. Normalized samples (30 μg/lane; Bio-Rad protein assay) underwent standardized electrophoresis in 10% SDS-PAGE containing 0.1% gelatin (Bio-Rad). Molecular weight markers (6-210 kDa), recombinant human latent MMP9 (92 kDa), and active MMP9 (83 kDa; Calbiochem) were loaded as positive controls. The gel was washed in 2.5% TX-PBS to remove SDS and renature the proteins, incubated at 37 °C for 48 h in an activation buffer (50 mM Tris-HCl, 200 mM NaCl, 10 mM CaCl_2_; pH 7.5), rinsed in ddw, and finally stained for 60 min with 0.25% Coomassie brilliant blue R250 in 40% isopropanol. Gelatinolytic activity was identified as clear bands on a uniform blue background following destaining in 7% acetic acid, indicating the area where gelatin was digested.

### Statistics

All experiments were done three times, with each point carried out in duplicate. Experimental results are expressed as mean±SEM. Parametric ANOVA followed by Tukey-Kramer post hoc was employed to analyze the data [19], using the statistical package StatView II for PC (Abacus Concepts Inc., Barkley, CA). A probability of less than or equal to 0.05 was considered to be statistically significant.

## Results

### Vernal keratoconjunctivitis-derived fibroblasts express nerve growth factor, trkA^NGFR^, p75^NTR^, and αSMA

**Figure 1:**
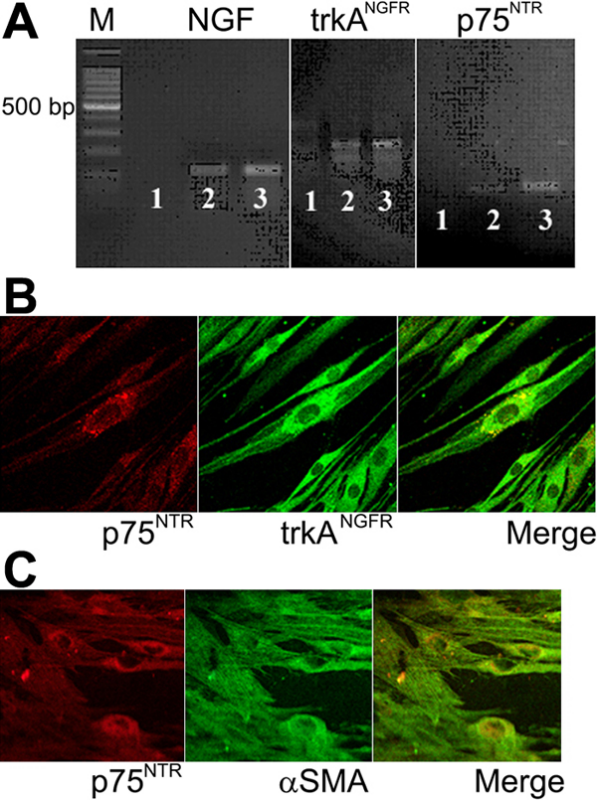
Nerve growth factor, trkA^NGFR^, p75^NTR^ and αSMA expression by vernal keratoconjunctivitis-derived fibroblasts. **A**: Conventional RT-PCR. showing from left to right, nerve growth factor (NGF; 120 bp), trkA^NGFR^ (103 bp); p75^NTR^ (100 bp) amplicons. The lines are as follows: (1) -RT, (2) healthy-FBs, and (3) VKC-FBs. These are representative gels from three independent experiments where equal amounts of cDNA were amplified. **B**: Confocal microscopy on VKC-FBs showing, from left to right, that VKC-FBs express p75^NTR^ (Cy3, red) and trkA^NGFR^ (Cy2, green). p75^NTR^ and trkA^NGFR^ colocalized in some cellular compartments (merge; X600/oil immersion). **C**: Confocal microscopy on VKC-FBs showing, from left to right, that these cells express p75^NTR^ (Cy3, red) and αSMA (Cy2, green). p75^NTR^ and αSMA colocalized in some cellular compartments (merge; X600/oil immersion).

Increased NGF levels were detected by specific ELISA in the conditioned media of VKC-FBs and compared to healthy-FBs (563.33±35.12 pg/ml versus 445.67±10.59 pg/ml; 26.51% increase; p=0.005). This data is in accordance with the NGF mRNA upregulation observed in VKC-FBs (Figure 1A). RT-PCR and confocal analysis revealed that VKC-FBs expressed both trkA^NGFR^ and p75^NTR^. In contrast, healthy FBs only express trkA^NGFR^, as previously reported [8] (Figure 1A,B). A slight downregulation of trkAmRNA expression was found in VKC-FBs, in comparison to healthy ones (p<0.05). As detected by cs-ELISA, a significant increase of αSMA expression was observed in VKC-FBs (0.378±0.027 OD VKC-FBs versus 0.232±0.046 OD healthy-FBs; a 1.62-protein increase [p<0.01]). VKC-FBs expressed αSMA protein in association with p75^NTR^ (Figure 1C). No proliferation was observed in the presence of increasing NGF concentrations (data not shown).

### Nerve growth factor does not modulate αSMA expression

**Figure 2:**
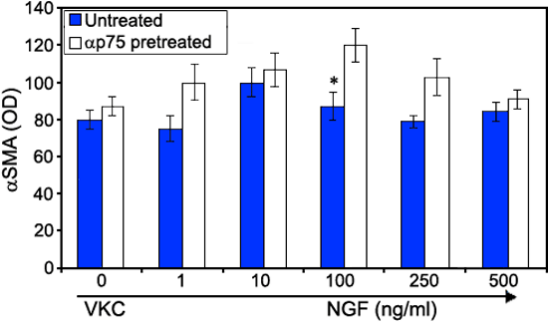
αSMA expression by vernal keratoconjunctivitis-derived fibroblasts. Increasing NGF concentrations did not influence αSMA expression by VKC-FBs (dark bar; p>0.05). αp75^NTR^ neutralization (1 μg/ml/30 min) significantly increased αSMA expression by NGF-treatment (light bar; p<0.05). αtrkA^NGFR^ neutralization did not influence αSMA expression by NGF treated VKC-FBs (data not shown). Representative data from three independent experiments, which results are shown as mean OD±SEM.

VKC-FBs were exposed to increasing NGF concentrations (1-500 ng/ml) in order to investigate whether NGF was able to influence αSMA expression. At both cs-ELISA and western blot analysis, NGF was not able to influence αSMA expression in VKC-FBs (Figure 2; dark bars [p>0.05]). NGF stimulation did not influence αSMA expression after preincubations with neutralizing trkA^NGFR^ antibodies, (data not shown). In contrast, preincubation with neutralizing p75^NTR^ antibodies followed by increasing NGF concentrations resulted in an increase of αSMA expression (Figure 2; light bars [p<0.05]). The pattern of αSMA expression resembled those of NGF-treated healthy FBs [8].

### Nerve growth factor modulates types I and IV collagen expression

**Figure 3:**
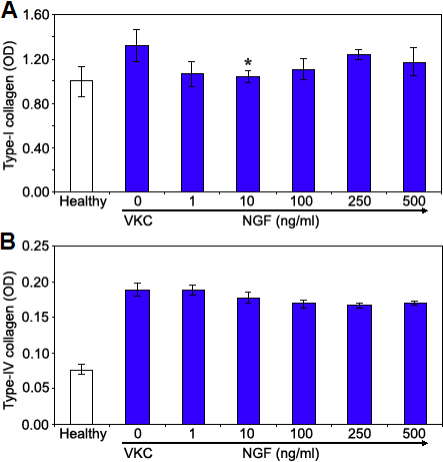
Nerve growth factor modulates type I and type IV collagen expression. VKC-FBs showed high types I and IV collagen production in comparison to healthy-FBs (rate: 7.78; p<0.05). Increasing NGF concentrations significantly decreased type I collagen expression (**A**), with a maximum at 10 ng/ml NGF (1.33 fold decrease; p<0.05). No effect was observed on type IV collagen expression (**B**). Representative data from three independent experiments are shown as mean OD±SEM.

In VKC-FBs, the high amount of αSMA was found associated with a high rate of types I and IV collagen (rate; 7.78: type I, 1.323±0.248 and type IV, 0.170±0.004), as compared to healthy FBs (type I, 0.993±0.001 and type IV, 0.077±0.014; p<0.05; see also Table 1). After NGF exposure, the expression of type I collagen by VKC-FBs was significantly decreased at all the concentration levels tested (Figure 3A). The maximum effect was observed at 10 ng/ml (1.33-target gene decrease, p<0.05), reaching a level almost comparable to those of healthy-FBs (Figure 3A; light bar). By contrast, the expression of type IV collagen was not changed at any NGF concentration level (Figure 3B; light bars).

**Table 1:** Biochemical and molecular description of healthy and vernal keratoconjunctivitis-derived fibroblasts.

**Experimental group**	Type-I collagen*	Type-IV collagen*	αSMA**	MMP9**	TIMP1**
Healthy (n=3)	0.993±0.001	0.077±0.014	3.60±0.70	8.01±0.22	8.70±0.12
VKC (n=3)	1.323±0.248	0.170±0.004	2.24±1.67	6.08±0.78	12.82±0.9S
*Analysis*	*+33%*	*+120%*	*+2.57*	*+3.81*	*-5.69*

Biochemical and molecular description of healthy and vernal keratoconjunctivitis-derived fibroblasts.

### Nerve growth factor modulates MMP-9 expression and function

**Figure 4:**
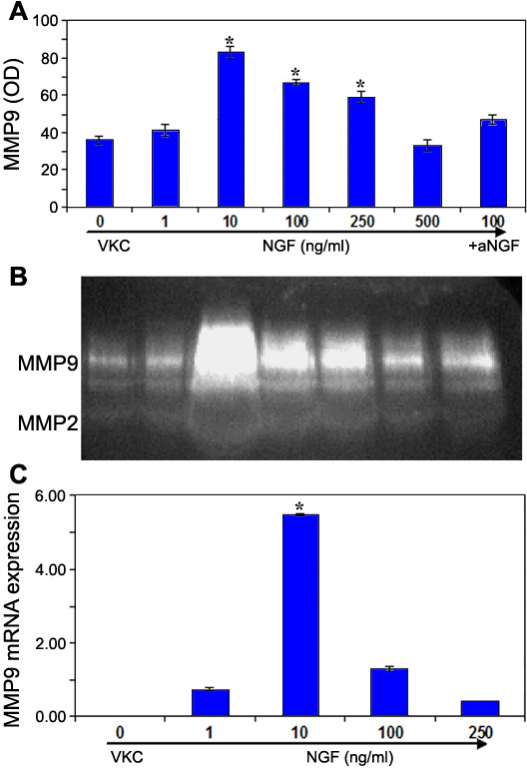
Nerve growth factor increases MMP9 expression and function by vernal keratoconjunctivitis-derived fibroblasts. Conditioned media were collected and processed as described in Methods. **A**: The histogram shows a significant increase of MMP9 protein expression in VKC-FBs treated with increasing NGF concentrations, according to the densitometric analysis (mean OD±SEM; p<0.05). Data were normalized to GAPDH expression and presented as fold increase with respect to untreated VKC-FBs. **B**: The functional activity in MMP9 was investigated by SDS-PAGE zymography. From left to right (1-6 lines): 0, 1, 10, 100, 250, 500 ng/ml NGF; line 7, αNGF+100 ng/ml NGF. Panel represents one of three independent gels that gave the same results. **C**: Relative real-time PCR showed a significant increase of MMP9 mRNA expression in VKC-FBs treated with different concentrations of NGF (p<0.05). Data were normalized to GAPDHmRNA expression and presented as fold increase [11] with respect to untreated VKC-FBs.

The expression and functional activities of MMP9, known to drive specifically collagen type IV cleavage [20], was investigated as a function of NGF stimulation in VKC-FBs. VKC-FBs were exposed to increasing NGF concentrations (1-100 ng/ml) and the expression of MMP9 protein and mRNA were evaluated and compared to untreated VKC-FBs. By western blot analysis, it was determined that NGF significantly increased MMP9 protein expression in VKC-FBs, as compared to healthy-FBs, in a dose-dependent fashion (Figure 4A). This specific MMP9 increase was associated with an increase of MMP9 activities, as shown by gelatinolytic bands, at the same concentrations tested (Figure 4B). In agreement with biochemical data, the molecular analysis showed that NGF was able to trigger the MMP9-mRNA expression in a dose-dependent fashion with the maximum expression at 10 ng/ml NGF (3.92-target gene increase, p<0.05; Figure 4C). Interestingly, the MMP9-mRNA increasing effect of 10 ng/ml NGF was found to be comparable to that of 1 ng/ml TGFβ1 (5.48±0.04 versus 7.49±0.09, respectively). According to the literature, MMP9 mRNA was found to be increased in VKC-FBs as compared to healthy-FBs (24.98-target gene increase; p<0.05 [3]). Biochemical and molecular characteristics of healthy and VKC-derived FBs are summarized in Table 1.

## Discussion

Our data demonstrated that primary cultures of VKC-FBs express both trkA^NGFR^ and p75^NTR^ receptors and produce high levels of NGF, types I and IV collagens, and MMP9. Moreover, VKC-FBs are mainly represented by myoFBs, since a consistent proportion of VKC-FBs were αSMA positive.

Various Th2-derived cytokines and growth factors are increased in blood, tears, and conjunctiva from VKC patients. These profibrotic factors were found able to drive the cross-talk between structural (epithelium and FBs) and inflammatory (lymphocytes, eosinophils, and mast cells) cells. This cell-factor interaction contributes to the chronic inflammatory process, giant papillae formation and tissue remodeling, as observed in VKC [2,3,5,21-23]. Among these, TGFβ1 isoform remains the main pro-fibrogenic factor, being responsible for ECM ex-novo deposition, the inhibition of ECM degradation and the prolonged myoFB activity [24].

NGF is also increased in VKC blood and conjunctiva as a result of the activation of both structural (epithelium and FBs) and inflammatory cells (Th2 lymphocytes, mast cells, and eosinophils), during the active conjunctivitis [7,22]. Older and more recent data [8,22-24] indicate that NGF is a pleiotrophic factor participating to the control of inflammatory responses, tissue repair, fibrosis, and remodeling in different tissues. Primary cultures of healthy-FBs have been found to be modulated by NGF with relation to cell migration, differentiation, and contraction of a cell matrix [8].

Since stromal FBs represent the major target/effector cells involved in tissue remodeling [6,12,25] and since NGF activates in vitro healthy-FBs [8,26,27], we sough to evaluate the possible modulation of FBs isolated from conjunctiva of patients with VKC by NGF, hence no data are available in literature.

In the present study, increased expression of NGF, associated with increased expression of p75^NTR^ and αSMA, was detected in VKC-FBs as compared with conjunctival healthy-FBs. In addition, αSMA was found expressed in FBs showing light upregulation of trkA^NGFR^ and considerable upregulation of p75^NTR^, suggesting a specific role for NGF in VKC-FBs. In line with the effect of NGF in driving the differentiation of healthy FBs into myoFBs [8], we wondered whether NGF supplementation to VKC-FBs cultures would result in a further VKC-FB differentiation, evaluated as αSMA expression. Interestingly, NGF failed to further increase αSMA expression in VKC-FBs, unless p75^NTR^ was blocked (see Figure 2; light bars). This data is of great interest, since it suggests two possible hypothesis: (1) NGF plays a differentiating effect through the specific and unique binding to trkA^NGFR^ (NGF is a specific receptor), but not p75^NTR^ (the pan-neurotrophin receptor); and (2) the specific expression of p75^NTR^, by myoFB phenotype (otherwise absent in FBs), seems to play a switch-off effect in the further differentiating action of NGF, suggesting that NGF plays a modulatory rather than a exclusive stimulatory effect on the fibrotic process. The answer might lie in trkA^NGFR^/p75^NTR^ multifaceted functions [7,22].

On the other side in VKC tissue remodeling process, NGF might modulate collagen production and might influence MMP2/MMP9 production/activity, as previously demonstrated for other growth factors [2,4]. ECM metabolism is heavily impaired in VKC, due to a substantial increase in total collagen deposition in the conjunctiva (mainly types I, III, and IV), and an increased release of MMP2/MMP9 in the tears [3,4]. In this study, the expression of type-I collagen decreased significantly after NGF exposure, rather quite specific given that this effect was not observed for type-IV collagen or with the addition of TGFβ1 (data not shown). In addition, NGF was able to induce specifically MMP9 expression/activity by VKC-FBs at both molecular, biochemical and functional levels. This effect was not observed in healthy-FBs, as previously reported [8].

Taken together, these data suggest a many-sided role of NGF in VKC tissue remodeling. NGF is increased in VKC blood and tarsal conjunctiva and likewise in tears [9]. In in vitro studies, NGF induces the differentiation of healthy conjunctival FBs into myoFBs, the main effector and target cells of fibrotic process. This differentiating effect was not observed in VKC-derived conjunctival FBs in the present study. Additionally, NGF induced a decrease in type I collagen and an increase in MMP9 expression by VKC-FBs with no specific effect on both MMP1 and MMP2 mRNA expression. It has been previously reported that both MMP2 and MMP9 degrade types IV and V collagen; MMP9 can also degrade types I and type III collagen [28,29]. One possible explanation for these data might be related to the well-known singular action of NGF due to changes in the balance between trkA^NGFR^/p75^NTR^ expression. In this study we demonstrated a role of trkA^NGFR^ in the differentiating effect of NGF on FBs; however, the role of p75^NTR^ on VKC-FB function seems complex and remains to be elucidated.

In VKC, various growth factors and Th2-cytokines are produced by inflammatory/stromal cells. In line with other studies, these factors have been proposed to modulate tissue remodeling in VKC. To summarize, our in vitro findings showing the NGF modulation of both type I collagen and MMP9, might propose NGF as an active contributor in VKC remodeling.
